# The lung function laboratory to assist in the management of chronic kidney disease

**DOI:** 10.36416/1806-3756/e20250094

**Published:** 2025-05-21

**Authors:** José Alberto Neder, Denis E O’Donnell, Danilo C Berton

**Affiliations:** 1. Pulmonary Function Laboratory and Respiratory Investigation Unit, Division of Respirology, Kingston Health Science Center & Queen’s University, Kingston (ON) Canada.; 2. Unidade de Fisiologia Pulmonar, Hospital de Clínicas de Porto Alegre, Universidade Federal do Rio Grande do Sul, Porto Alegre (RS) Brasil.

## BACKGROUND

The kidneys and lungs share the mutual task of maintaining acid-base homeostasis. Abnormalities in fluid balance, blood electrolytes, hemoglobin concentration (Hb), and vascular tone in chronic kidney disease (CKD) may have profound effects on respiratory function. Chronic lung disease and CKD frequently coexist,^1^ creating further challenges to pulmonary function tests (PFTs) interpretation. 

## OVERVIEW

A 68-year-old man under long-term hemodialysis (HD) and progressive exertional dyspnea underwent PFTs as part of the workup pre-kidney transplant. Spirometry suggested, and body plethysmography confirmed, mild restriction. Hb-corrected DL_CO_ and K_CO_ were severely reduced; further workup confirmed mixed pre- and post-capillary pulmonary hypertension (PH). Despite comprehensive treatment, the patient died before transplantation (**patient A**). A 77-year-old woman with “mild” COPD on spirometry presented with severe hypoxemia (¯ PaO_2_) but only mild-moderate decrements in SpO_2_ ~two hours within the HD sessions. Detailed assessment of gas exchange in a subsequent session showed similar decrements in the alveolar pressure for O_2_ (PAO_2_) and a measured respiratory exchange ratio (RER; CO_2_ output/O_2_ consumption) of only 0.63 (0.85 pre-HD). She was diagnosed with HD-induced hypoxemia (**patient B**).

**Figure 1:**
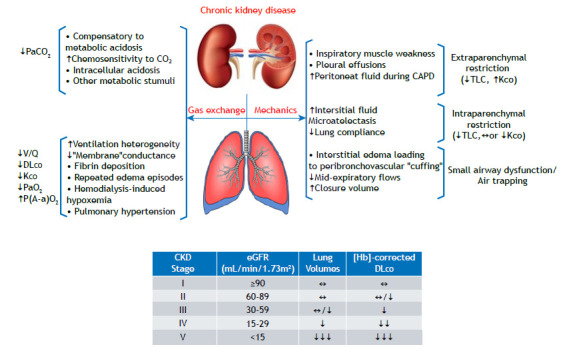
A simplified overview of the main respiratory consequences of chronic renal failure and their impact on common pulmonary function tests. The table depicts the relationship between chronic kidney disease (CKD) progression towards failure and the most relevant respiratory functional findings in stable patients. Abbreviations: PaCO_2_: partial arterial pressure of carbon dioxide; V/Q: (alveolar) ventilation/perfusion ratio; K_CO_: carbon monoxide transfer coefficient; PaO_2_: partial arterial pressure of oxygen; P(A-a)O_2_: alveolar-arterial oxygen pressure difference; CAPD: continuous ambulatory peritoneal dialysis; eGFR: estimated glomerular filtration rate; ¯: decreased; ­: increased; and «: preserved.

CKD patients often develop a mild restrictive spirometry pattern, which has been traditionally ascribed to chronic fluid overload. They show an exquisite sensitivity to develop “flash” interstitial edema over time, causing repeated episodes of acute-on-chronic restriction. However, lung volumes may decrease without overt edema, likely reflecting interstitial structural abnormalities and low compliance.^2^ When the glomerular filtration rate decreases further, pulmonary edema, pleural effusion, and respiratory muscle dysfunction are more common. Electrolyte disturbance may also contribute to skeletal (including respiratory) muscle weakness. In some patients, apparent restriction on spirometry is not due to low TLC but higher residual volume, likely due to early closure of the dependent small airways (Figure 1).^3^

Anemia is a major determinant of low DL_CO_ (and K_CO_) in CKD. Interestingly, both may remain low after Hb correction. This has been ascribed to lower “membrane” conductance rather than impaired capillary blood flow.^4^ It remains present after dialysis and may not completely reverse after kidney transplantation.^2^ The underlying mechanisms are unclear, but akin to heart failure, it may result from the deposition of fibrin following repeated episodes of clinical or sub-clinical edema. Low DL_CO_ may also signal PH, a comorbidity closely associated with poor prognosis (**patient A**).

Some patients-particularly those with underlying lung disease-may develop significant hypoxemia as the HD session progresses. Several mechanisms have been put forward; most evidence points out the loss of CO_2_ into the dialysis fluid. The non-respiratory loss of CO_2_ causes a lower CO_2_ output at the mouth, leading to hypoventilation and a lower RER. Since PAO_2_ = PiO_2_ - (PACO_2_/RER), a low RER implies lower PAO_2_ and, consequently, lower PaO_2_.^5^ A shift in the O_2_ dissociation curve due to the increasing pH explains why SpO_2_ may underestimate the severity of hypoxemia (**patient B**). 

## CLINICAL MESSAGE

Most centers recommend “full” PFTs pre-kidney transplantation for higher-risk patients, i.e., dialysis for an extended period, diagnosis of COPD, history of tobacco exposure, obstructive sleep apnea, previous pulmonary embolism, and/or suspected PH.^6^ Close interaction between nephrologists and pulmonologists is paramount, given the bidirectional nature of the observed abnormalities in a population that may present with preexisting, frequently underrecognized lung dysfunction.

## References

[1] Navaneethan SD, Mandayam S, Arrigain S, Rahman M, Winkelmayer WC, Schold JD (2016). Obstructive and Restrictive Lung Function Measures and CKD National Health and Nutrition Examination Survey (NHANES) 2007-2012. Am J Kidney Dis.

[2] Sidhu J, Ahuja G, Aulakh B, Narang A, Whig J, Sidhu U (2007). Changes in pulmonary function in patients with chronic renal failure after successful renal transplantation. Scand J Urol Nephrol.

[3] Gembillo G, Calimeri S, Tranchida V, Silipigni S, Vella D, Ferrara D (2023). Lung Dysfunction and Chronic Kidney Disease A Complex Network of Multiple Interactions. J Pers Med.

[4] Moinard J, Guenard H (1993). Membrane diffusion of the lungs in patients with chronic renal failure. Eur Respir J.

[5] Patterson RW, Nissenson AR, Miller J, Smith RT, Narins RG, Sullivan SF (1981). Hypoxemia and pulmonary gas exchange during hemodialysis. J Appl Physiol Respir Environ Exerc Physiol.

[6] Sahni S, Molmenti E, Bhaskaran MC, Ali N, Basu A, Talwar A (2014). Presurgical pulmonary evaluation in renal transplant patients. N Am J Med Sci.

